# Anti-Inflammatory Effect of Phytoncide in an Animal Model of Gastrointestinal Inflammation

**DOI:** 10.3390/molecules26071895

**Published:** 2021-03-26

**Authors:** Azra Memon, Bae Yong Kim, Se-eun Kim, Yuliya Pyao, Yeong-Geun Lee, Se Chan Kang, Woon Kyu Lee

**Affiliations:** 1 Department of Biomedical Sciences, School of Medicine, Inha University, Incheon 22212, Korea; azrabiochem@yahoo.com (A.M.); julipyao@gmail.com (Y.P.); 2 Research Institute, Phylus Co., LTD., Danyang-gun 27000, Korea; kby2974@hanmail.net; 3 Department of Oriental Medicine Biotechnology, College of Life Sciences, Kyung Hee University, Yongin-si 17104, Korea; tpdms83@hanmail.net (S.-e.K.); lyg629@nate.com (Y.-G.L.); sckang@khu.ac.kr (S.C.K.)

**Keywords:** *Pinus koraiensis*, phytoncide, anti-inflammation, gastritis, colitis

## Abstract

*Background:* Phytoncide is known to have antimicrobial and anti-inflammatory properties. *Purpose:* This study was carried out to confirm the anti-inflammatory activity of two types of phytoncide extracts from pinecone waste. *Methods:* We made two types of animal models to evaluate the efficacy, an indomethacin-induced gastroenteritis rat model and a dextran sulfate sodium-induced colitis mouse model. *Result:* In the gastroenteritis experiment, the expression of induced-nitric oxide synthase (iNOS), a marker for inflammation, decreased in the phytoncide-supplemented groups, and gastric ulcer development was significantly inhibited (*p* < 0.05). In the colitis experiment, the shortening of the colon length and the iNOS expression were significantly suppressed in the phytoncide-supplemented group (*p* < 0.05). *Conclusions:* Through this study, we confirmed that phytoncide can directly inhibit inflammation in digestive organs. Although further research is needed, we conclude that phytoncide has potential anti-inflammatory properties in the digestive tract and can be developed as a functional agent.

## 1. Introduction

Gastritis is an inflammation, irritation, or erosion of the lining of the stomach. It can be caused by irritation due to excessive alcohol use, chronic vomiting, stress, or the use of anti-inflammatory drugs. The best-known examples of such medications are non-steroidal anti-inflammatory drugs (NSAIDs). NSAIDs are some of the most widely used drugs in the world [1]. Gastrointestinal damage is known to be a common adverse effect of NSAIDs, and it results in outcomes ranging from nonspecific abdominal pain to ulceration, hemorrhage, and death [2]. This effect generally correlates with inhibition of cyclooxygenase (COX) [3] the key enzyme in prostaglandin (PG) synthesis [4,5]. Decreased PG synthesis leads to gastrointestinal damage by reducing mucosal blood flow, mucus secretion, bicarbonate production, epithelial cell turnover, and wound healing [6,7,8,9], while activated neutrophils release reactive oxygen species (ROS) that contribute to gastric mucosal microcirculatory disorder. Most NSAIDs (including acetylsalicylic acid) remain in their non-ionized lipophilic form in the highly acidic gastric environment, enabling them to migrate into surface epithelial cells. The NSAIDs are then dissociated into their ionized form, which traps hydrogen ions, which in turn induces mucosal injury [10] thereby increasing the risk of attack by endogenous [11] and exogenous factors. Additionally, the inhibition of COX-1 blocks platelet production of thromboxane, which increases bleeding [12]. Thus, NSAIDs can adversely affect both the upper and lower gastrointestinal tract. Indomethacin (IM) is a representative NSAID that also has antipyretic, antithrombotic, and analgesic effects. As a non-steroidal drug, it has a tendency to cause gastrointestinal tract damage, such as stomach ulcers [13]. Therefore, IM is widely applied in scientific research trials to induce gastritis in animals.

Inflammatory bowel diseases (IBD) are classified as refractory intestinal diseases that include Crohn’s disease and ulcerative colitis (UC). IBD can be characterized by chronic inflammation involving mucosal erosions and ulcers in the gastrointestinal tract [14]. In the following study, we focus on ulcerative colitis, which can be characterized by body weight loss, tissue disruption with relapsing bloody diarrhea, intestinal barrier dysfunction with inflammatory cell infiltration [15], and sores on the inner lining of the digestive tract, mainly in the large intestine [16]. In accordance with the recent evidence, the disease is caused by a combination of factors, including genetics, barrier dysfunction, change in microbial flora, and immune dysregulation. Commonly, UC is associated with increased production of inflammatory mediators including ROS, nitric oxide (NO), and PG, and upregulation of certain proteins, i.e., COX-2 or inducible nitric oxide synthase (iNOS), that play a role in dysregulation of the immune system. Furthermore, upregulated production of a complex mixture of inflammatory mediators [17] and abnormal cell influx into the inflamed intestine are considered prominent histopathologic features of IBD [18]. In the majority of the experiments, dextran sodium sulfate (DSS) has been exploited to produce an animal model of colitis. DSS is a synthetic sulfate polysaccharide that induces UC in rodents due to altered colonic microflora or macrophage activity or direct toxicity to crypt cells [19]. DSS can initiate immense macrophage and granulocyte infiltration [20] and can cause the upregulation of pro-inflammatory cytokines and chemokines (IL-1, IL-6, keratinocyte-derived cytokine, tumor necrosis factor-α, and interferon-γ) [21,22]. Oral administration of DSS induces colitis resembling UC in hamsters and mice [23]. Due to its simplicity and high degree of uniformity and reproducibility of most lesions in the distal colon [24,25], DSS-induced colitis in mice is a well-accepted and widely used IBD animal model [26].

Phytoncides are known as antimicrobial, volatile organic compounds derived from plants as a natural antibacterial substance. The word phytoncide can be translated as ‘exterminated by the plant’. Phytoncide solutions have a variety of components, including volatile terpenoids such as α-pinene, carene, and myrcene [27]. The most significant amongst these terpenoids is a monoterpenoid called α-pinene, which is responsible for the anti-inflammatory effect of phytoncide [27]. Furthermore, phytoncide has antimicrobial, antioxidant and anti-cancer activities [28,29]. It was reported that phytoncide significantly enhances human natural killer cell activity [29].

The objective of this study was to elucidate if phytoncide can heal mucosal damage in the digestive tract, namely in the stomach and to show phytoncide’s anti-inflammatory influence on an animal’s digestive tract, which could be considered a first discovery which could possibly lead to use of phytoncide as a natural material in anti-inflammatory medicines in the future.

## 2. Materials and Methods

### 2.1. Phytoncide

#### 2.1.1. Preparation of Pine Cone Extracts

Pine cone waste from *Pinus koraiensis* was purchased from Gapyeong, Gyeonggi-Do, Korea. A voucher specimen (voucher # IH-01-035) was deposited at the herbarium of Inha University (Incheon, Korea). Phytoncide extracted from pine cone waste was provided by PHYLUS (Danyang, Chungcheongbuk-Do, Korea). Phytoncide extracts were prepared using the steam distillation method described by [30]. First, pine nuts were blown off and pine cone wastes with a water content of 28% were dried in the shade to a final water content of 20%. After adjusting to 2–4 cm, 1 kg was placed in a 5 L round-bottomed flask, and the distillation temperature was maintained at 100 ± 3 °C and water vapor was passed directly to the bottom of the sample with a separate steam system. The solution obtained by distillation was separated into the oil phase and the water phase through the specific gravity difference. The oil phase was phytoncide oil (PO) and the water phase was pine cone water (PW), a hydrosol containing the water-soluble phytoncide components. The phases were separated, sealed and refrigerated until used in the test. Among the extracts, PW was formed by dissolving a water-soluble component of *P. koraiensis* phytoncide into water vapor supplied during the extraction process. Therefore, it is not highly volatile like PO, but it was expected to have the main functional material, and thus it was used as a research sample.

#### 2.1.2. Gas Chromatography-Mass Spectrometer Analysis of Extracts

Gas chromatography-mass spectrometer (GC-MS) analysis was performed on the compounds known to be present in phytoncide. The major components of the extract were separated using an HP-5MS Column (30 m × 0.25 mm, 0.25 μm, Agilent Technologies, Santa Clara, CA, USA), and detailed conditions are shown in Table 1. The identification of eight analytes was confirmed by comparing their spectra and retention times with standards from the library NIST 2.0. Quantitative analysis of α-pinene, which is known to have an anti-inflammatory effect among the compounds found in phytoncide extract, was measured as follows:

(1) $$\alpha -\mathrm{Pinene}\;(\mathrm{mg}/L)\;=\;\mathrm{Concentration}\;\mathrm{of}\;\mathrm{test}\;\mathrm{solution}\;\left(\mu g/\mathrm{mL}\right)\times \frac{\mathrm{Volume}}{\mathrm{sample}\;\mathrm{weight}\;\left(g\right)}\times \mathrm{standard}\;\mathrm{purity}$$

Analysis of each sample was performed three times.

## 3. Indomethacin-Induced Gastritis Rat Model

### 3.1. Chemicals

The indomethacin (IM, cat# 17378), lansoprazole (LP, cat# L8533), sodium bicarbonate (NaHCO_3_, cat# S5761) and carboxymethyl cellulose (CMC, cat# C5678) used in this experiment were obtained from Sigma-Aldrich (St. Louis, MO, USA). IM was suspended in 5% NaHCO_3_ and LP was liquefied in 0.5% CMC.

### 3.2. Animal Care

Six-week-old male Sprague–Dawley rats were purchased from Orient-Bio Corporation (Seongnam, Gyeonggi-Do, Korea) and acclimated for 1 week before the experiment. The rats were maintained under standard environmental conditions, with constant temperature (23 ± 3 °C) and humidity (53 ± 2%) under 12 h light/dark cycles. The rats were fed a standard diet and water ad libitum. Two rats were housed per cage to achieve an appropriate vigorous condition. All animal studies were approved by the Animal Research Committee of Inha University and were maintained according to the guidelines of the Animal Facility. Animal experiments were performed in accordance with the current ethical regulations for animal care and use at Inha University (IRB File No. 2018-11-020).

### 3.3. Experimental Design

The rats were divided into 7 groups of 10. For 24 h, all rats were fasted with free access to tap water. All chemicals and treatment solutions were administered via oral gavage. Two types of phytoncide extract were administered to four groups, at doses of PO 5 and 10 mL/kg and PW 5 and 10 mL/kg, respectively. LP was established as a positive control group at a dose of 30 mg/kg. First, LP and phytoncide extracts were provided by oral administration. After 30 min, 80 mg/kg of IM was orally administered to each rat in all experimental groups to induce gastric ulceration. The rats in the control group received distilled water (DW). The animals were sacrificed 6 h later using isoflurane solution (JW Pharmaceutical Corporation, Seoul, Korea).

### 3.4. Evaluation of Inflammation in Indomethacin-Induced Gastric Ulcers

Evaluation of inflammation in gastric ulcers was conducted by determining the mucosal hemorrhagic lesions and calculating the damaged ulcer area. The gastric mucosal lesions were calculated using an analysis system: an AxioVision 4.8 imaging analyzer (SPOT software, version 4.6). The Gastric ulcer score was calculated as the sum of the areas of all hemorrhagic lesions, using the following formula:

(2) $$\mathrm{Gastric}\;\mathrm{ulcer}\;\mathrm{score}\;\left(\%\right)=\;\frac{\mathrm{Total}\;\mathrm{lesion}\;\mathrm{area}}{\mathrm{Total}\;\mathrm{gastic}\;\mathrm{mucosal}\;\mathrm{area}}\times 100$$

### 3.5. Histological Evaluation

Animals’ stomachs were immediately removed and fixed with 5% neutral buffered formalin (NBF) for 10 min. Afterwards, each stomach was opened along the great curvature and gently washed with normal saline for assessment of gastric ulceration. Then, stomach tissue was immersed in 10% NBF and stored 24–48 h for further histopathological and immunohistochemistry analysis. Protective effects of phytoncide extracts were investigated by comparison with the results obtained from the vehicle group (G2).

Consequently, obtained paraffin blocks were transversally cut into 5-μm thick sections, mounted on glass slides and stained with hematoxylin & eosin (H&E, Sigma-Aldrich). Histologic evaluation was performed based on the following scoring system: (A) inflammation: no inflammation = 0; mild = 1; moderate = 2; severe = 3, (B) degree of ulceration: no ulcer = 0; 1–2 ulcers involving up to 20 crypts = 1; 1–4 ulcers involving up to 20–40 crypts = 2; 1–4 ulcers involving more than 40 crypts = 3, (C) hyperplasia of crypt: no crypts = 0; mild = 1; moderate = 2; severe = 3. Finally, the histological scores were assessed from 0 to 9 by summarizing the results of all 3 parameters of the scoring system described above.

### 3.6. Immunohistochemistry

The tissue samples embedded in paraffin block sections were sliced thinly into 3-μm width sections and dried at 60 °C. Each section was sequentially deparaffinized by differently concentrated xylene solutions (from 85% to 99.99%) for 10 min and hydrated by alcohol one by one, from 100% to 90% alcohol. Afterwards, sections were washed with DW, boiled in 0.1 M citrate buffer at pH 6.0 for 10 min to retrieve antigens and incubated in 3% hydrogen peroxide for 10 min at room temperature to block endogenous peroxidase activity. Finally, obtained samples were incubated at 4 °C overnight in a humidified chamber with iNOS rabbit polyclonal antibody (PA5-16855, Thermo Fisher Scientific, Waltham, MA, USA). EnVision + System-HRP Labelled Polymer Anti-Rabbit (K4002, Agilent Dako, Santa Clara, CA, USA) was used as a secondary antibody for 30 min at room temperature. For visualization, 3,3′-diaminobenzidine peroxidase substrate was applied. Counterstaining was performed with hematoxylin and water containing 3% ammonia. Ultimately, sections were dehydrated and cleared in xylene. These slides were examined under an optical microscope (Nikon, Tokyo, Japan) to estimate the inflammation damage. Immunohistochemical measurement was performed with SPOT Software (Version 4.6, Flex 4 MP Mosaic, Micro Video Instruments, Inc. Avon, MA, USA).

## 4. Dextran Sulfate Sodium-Induced Colitis Animal Model

### 4.1. Chemicals

Dextran sulfate sodium salt (DSS, colitis grade, cat# 160110) was purchased from MP Biomedicals (Solon, OH, USA). DSS was dissolved in DW and stirred until completely transparent.

### 4.2. Animal Care

Five-week-old male C57BL/6 mice were purchased from Orient-Bio Corporation and acclimated for 1 week before the experiment. The mice were maintained under standard environmental conditions, with constant temperature (23 ± 3 °C) and humidity (53 ± 2%) under 12 h light/dark cycles. They were given a standard diet and water ad libitum. The mice were housed 5 mice per cage to achieve an appropriate vigorous condition. All animal studies were approved by the Animal Research Committee of Inha University and were maintained according to the guidelines of the Animal Facility. Animal experiments were performed in accordance with the current ethical regulations for animal care and use at Inha University (IRB File No. 2018-11-020).

### 4.3. Experimental Design

The total duration of the experiment was 14 days. Sixty mice were allocated into 6 groups of 10 mice of matched age and sex. In the first group, designated as the normal control (G1), the mice received a normal diet and DW ad libitum. The second group, labeled as the DSS control (G2, vehicle), received 2% DSS dissolved in DW during the last 7 days. In the remaining four groups, administration was based on two types of approaches: prevention and therapy. The prevention method implied administration of treatment solutions throughout the whole experiment period, whereas in the therapy method, treatment solutions were given at the same days as 2% DSS. The two types of solutions of phytoncide extract (PO and PW with treatment dose: 10 mL/kg, volume: 200 µL) were administered to four groups of mice: 2% DSS + PO (prevention, G3); 2% DSS + PO (therapy, G4); 2% DSS + PW (prevention, G5); and 2% DSS + PW (therapy, G6), respectively. After 2 weeks from the beginning of the experiment, the mice continued to receive 2% DSS, and were fasted for 16 h before the end of the experiment.

The animals were anesthetized with zoletil (Virbac Korea, Seoul, Korea) and xylazine HCl (Sigma-Aldrich) (ratio 1:1, 0.05 mL/mouse) and their colons were excised. The colons were photographed and their lengths were measured. Dissected tissues were fixed in 10% NBF using the Swiss roll technique, and further processed for H&E staining and immunohistochemistry. The obtained slides were monitored under a light microscope to check the colonic damage.

### 4.4. Evaluation of Disease Activity Index (DAI)

To determine the clinical disease activity index (DAI), mice were examined daily starting from the day prior to colitis induction and throughout the experiment, considering the following parameters: body weight loss (scored as: none = 0; 1–5% = 1; 5–10% = 2; 10–15% = 3; above 15% = 4); stool consistency (scored as: normal = 0; soft = 2; diarrhea = 4), presence or absence of fecal blood (scored as: none = 0; little bleeding = 2; massive bleeding = 4). The clinical DAI score was estimated as the sum of the above parameters, calculated from 0 to 12. Two weeks later, colons were dissected. Differences between lengths in all groups were calculated.

### 4.5. Histological Evaluation

Dissected tissues were fixed by 10% NBF overnight, dehydrated (graded alcohols), cleared (xylene) and embedded into paraffin wax. Subsequently obtained blocks were cut into 5-μm thick transversal sections and mounted on glass slides, used for H&E staining. A histological scoring system was used to check the severity of inflammation damage, where features were graded as follows: (A) inflammation: no inflammation = 0; mild = 1; moderate = 2; severe = 3, (B) degree of ulceration: no ulcer = 0; 1–2 ulcers involving up to 20 crypts = 1; 1–4 ulcers involving up to 20–40 crypts = 2; more than 40 = 3, (C) hyperplasia of crypt: no crypts = 0; mild = 1; moderate = 2; severe = 3. The summation of these scores (0 to 9) reflected actual inflammation damage.

### 4.6. Immunohistochemistry

The tissue samples embedded in paraffin block sections were sliced thinly into 3-μm width sections and dried at 60 °C. Each section was sequentially deparaffinized with xylene solutions of different concentrations (from 85% to 99.99%) during 10 min and hydrated by alcohol one by one, from 100% to 90% alcohol. The sections were washed with DW, boiled in 0.1 M citrate buffer with pH 6.0 for 10 min to retrieve antigens and incubated in 3% hydrogen peroxide for 10 min at room temperature to block endogenous peroxidase activity. Finally, obtained samples were incubated at 4 °C overnight in a humidified chamber with iNOS rabbit polyclonal antibody (PA5-16855, Thermo Fisher Scientific). EnVision + System-HRP Labelled Polymer Anti-Rabbit (K4002, Agilent Dako) was the secondary antibody for an incubation of 30 min at room temperature. For visualization, 3,3′-diaminobenzidine peroxidase substrates were applied. Counterstaining was performed by hematoxylin and water containing 3% ammonia. Ultimately, sections were dehydrated and cleared in xylene. These slides were examined under a light microscope to estimate the inflammation damage. Immunohistochemical measurement was performed with SPOT Software (Version 4.6, Flex 64 MP Mosaic camera).

## 5. Statistical Analysis

All parameters were expressed as mean ± standard error of the mean (SEM). Statistical analyses of the experiment were performed using one-way analysis of variance, followed by Tukey’s multiple comparison methods or a Kruskal–Wallis test. A reliable probability of *p* < 0.05 was regarded as statistically significant. Statistical calculations were performed with SPSS software for Windows (Version 21.0; SPSS, IBM, Armonk, NY, USA).

## 6. Results

### 6.1. GC-MS Analysis of Phytoncide Extract

We performed a GC-MS analysis to identify the major compounds in the phytoncide extract. As shown in Table 2, α-pinene, camphene, β-pinene, β-myrcene, bicyclo(4.1.0)hept-3-ene, limonene, 1,4-methanoazulene and caryophyllene were present. α-Pinene was the main compound at 360,819 mg/L.

### 6.2. Indomethacin-Induced Gastritis Rat Model

#### 6.2.1. Phytoncide Treatment Ameliorates the Severity of Gastric Ulcers in Indomethacin-Induced Gastric Inflammation

**Figure 1:**
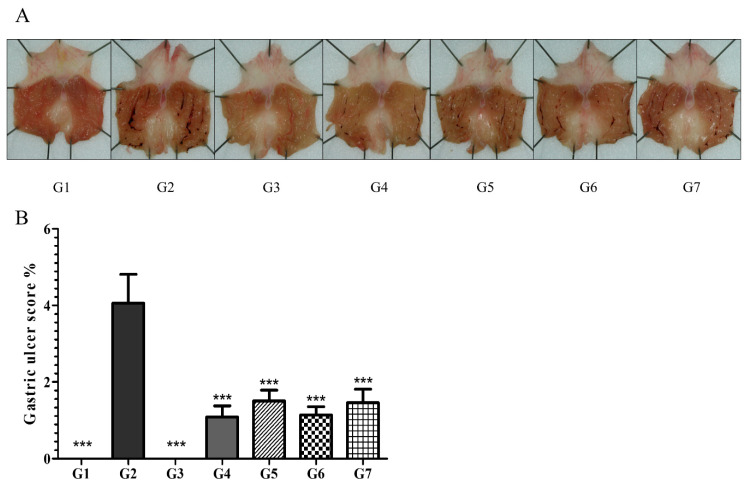
Effect of phytoncide on an indomethacin-induced gastric ulcer. (**A**) Photograph of the stomach of a rat with indomethacin-induced gastritis, (**B**) gastric ulcer scoring graph of a rat with indomethacin-induced gastritis. G1, normal control; G2, Vehicle control (gastric ulcer control, indomethacin 80 mg/kg); G3, positive control (lansoprazole 30 mg/kg); G4, phytoncide oil 10 mL/kg; G5, phytoncide oil 5 mL/kg; G6, pine cone water extract 10 mL/kg; G7, pine cone water extract 5 mL/kg. Representative data are expressed as mean ± SEM of experiments (*n* = 10) for each group. *** *p* < 0.001 compared to vehicle control.

The effects of phytoncide on gastropathy with IM-induced gastric ulcers was determined by an optical microscope and calculated with a computer-assisted analysis system. IM at a dose of 80 mg/kg induced severe lesions in the stomach mucosa. The appearance of lesions was validated by clear visibility of almost black-colored spots and linear portions, especially in vehicle controls (G2), where ulceration was more marked. Gastritis severity was significantly lower in groups from G4 to G7. The result confirmed that phytoncide administration led to a suppression of gastritis development. This suppression was more marked in the groups that were administered 10 mL/kg (G4, G6) than in those administered 5 mL/kg (G5, G7) (Figure 1).

#### 6.2.2. Phytoncide Inhibits Histological Changes in Indomethacin-Induced Gastric Inflammation

**Figure 2:**
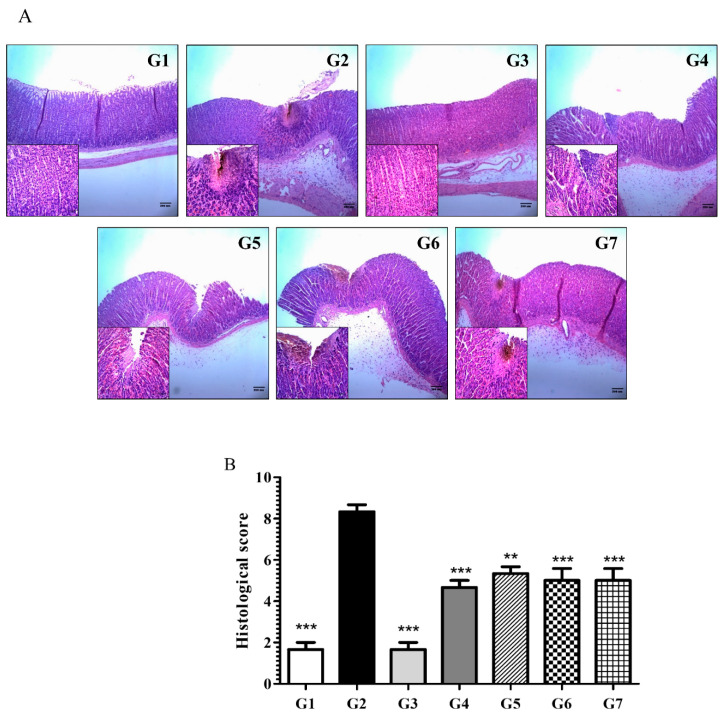
Effect of phytoncide treatment on histological changes in indomethacin-induced gastric damage. (**A**) Results of the H&E staining, × 100 magnification. (**B**) Effect of phytoncide on histological scores of the indomethacin-induced gastric ulcer. G1, normal control; G2, vehicle control (gastric ulcer control, indomethacin 80 mg/kg); G3, positive control (lansoprazole 30 mg/kg); G4, phytoncide oil 10 mL/kg; G5, phytoncide oil 5 mL/kg; G6, pine cone water extract 10 mL/kg; G7, pine cone water extract 5 mL/kg. Representative data are expressed as mean ± SEM of experiments (*n* = 10) for each group. ** *p* < 0.01 and *** *p* < 0.001 compared to vehicle control.

Samples were stained with H&E and evaluated to observe the histological changes in stomach mucosa induced by IM administration. As shown in Figure 2, normal controls (G1) and positive controls (G3) tended to show similar results, with the gastric layers having a normal structure. Contrastingly, the disease controls demonstrated markedly clear ulceration and inflammation as well as infiltration and necrosis of cells of mucosa (G2). The groups that were receiving phytoncide through oral administration (G4–G7) showed distinct recovery in terms of these parameters. Despite the general tendency towards diminished histological changes in all pretreated groups, their severity varied in a dose-dependent manner. Conclusively, the evaluation of microphotographs confirmed that phytoncide treatment could alleviate IM-induced gastric ulceration in rats. Calculation of the histological scores validated that phytoncide-treated groups (from G4 to G7) had maintained normal morphology of stomach cells, whereas those of disease controls had undergone noticeable destruction. Figure 2B is a graphical representation of these histological changes, showing that the IM-induced gastric ulceration was significantly inhibited in the phytoncide-treated groups.

#### 6.2.3. Phytoncide Suppresses iNOS Expression in Indomethacin-Induced Gastric Damage

**Figure 3:**
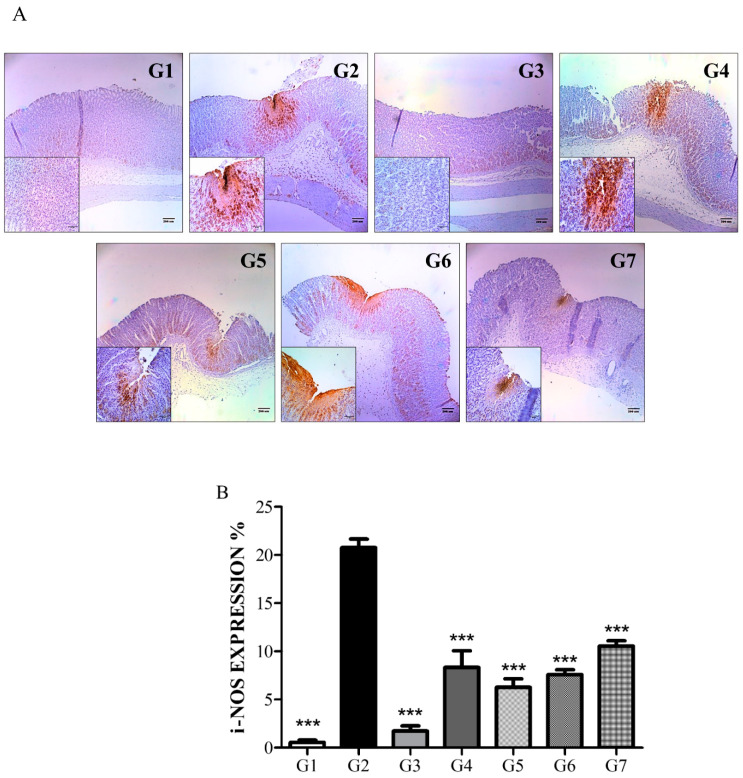
Immunohistochemical analysis: effect of phytoncide treatment on iNOS expression in indomethacin-induced gastric damage. (**A**) Photograph of stomach tissue from rat with indomethacin-induced gastritis, × 100 magnification. (**B**) iNOS expression graph of rat with indomethacin-induced gastritis. G1, normal control; G2, vehicle control (gastric ulcer control, indomethacin 80 mg/kg); G3, positive control (lansoprazole 30 mg/kg); G4, phytoncide oil 10 mL/kg; G5, phytoncide oil 5 mL/kg; G6, pine cone water extract 10 mL/kg; G7, pine cone water extract 5 mL/kg. *** *p* < 0.001 compared to vehicle control.

As a confirming evaluation procedure, the expression of iNOS, one of the common inflammatory markers, was measured. The level of iNOS expression was measured by immunohistological analysis of the brown-colored areas on the microphotographs (Figure 3). Predictably, the vehicle control group (G2) had the highest level of iNOS expression, thereby indicating a severe inflammatory process. The expression of iNOS in all the phytoncide-treated groups was prominently reduced, which verifies the inflammation-protective effects of phytoncide extracts.

### 6.3. DSS-Induced Colitis Animal Model

#### 6.3.1. Phytoncide Treatment Ameliorates Inflammatory Parameters in DSS-Induced Colitis

**Figure 4:**
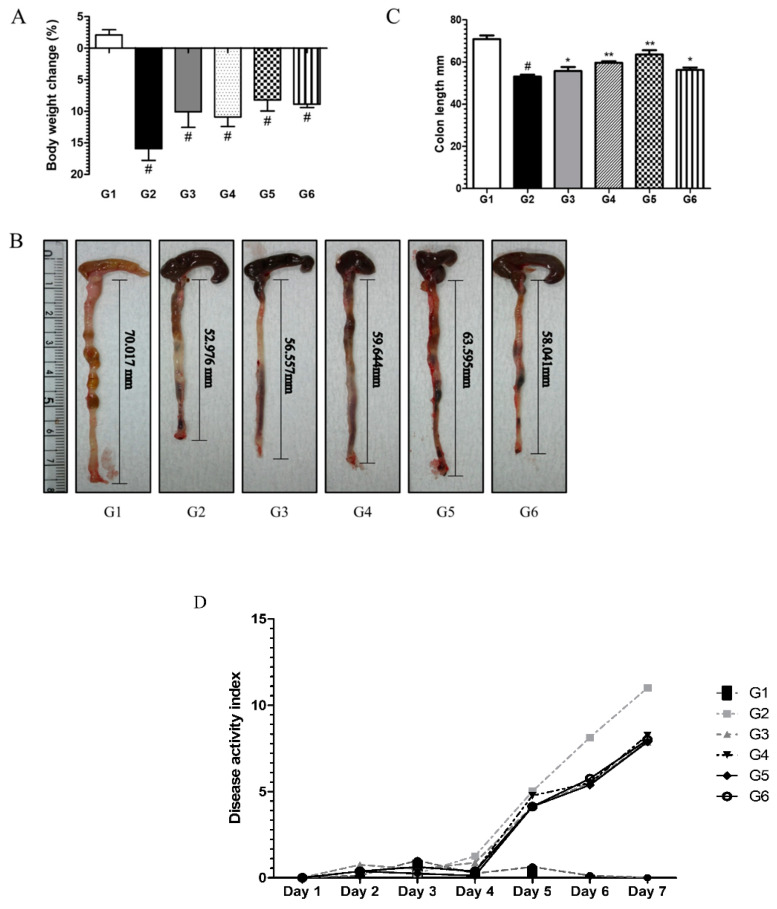
Effect of phytoncide treatment on symptoms of mice with DSS-induced colitis. (**A**) Effect of phytoncide treatment on body weight change in mice with DSS-induced colitis, (**B**) effect of phytoncide treatment in photograph of colon tissues of mice with DSS-induced colitis, (**C**) graphs of colon length, (**D**) effect of phytoncide treatment on disease activity index in mice with DSS-induced colitis. G1, normal control; G2, vehicle control (colitis control, DSS 2% in DW); G3, prevention phytoncide oil 10 mL/kg; G4, therapy phytoncide oil 10 mL/kg; G5, prevention pine cone water extract 10 mL/kg; G6, therapy pine cone water extract 10 mL/kg. Representative data are expressed as mean ± SEM of experiments (*n* = 10) for each group. ^#^*p* < 0.05 compare to normal control. * *p* < 0.05 and ** *p* < 0.01 compared to vehicle control.

During the first days of DSS administration, the mice showed signs of good health, such as normal well-formed stool and gradual increase in body weight. However, for the last two days of treatment, signs of inflammation increased rapidly. Prominent clinical changes such as occult blood, loose stool (data not shown), and body weight loss were observed in the disease control group (G2), while in the phytoncide pretreated groups (G3–G6), these symptoms occurred at a lower intensity. However, as shown in Figure 4A, body weight loss was notable in pretreated mice. The mice from the normal control group (G1) remained healthy throughout the experiment. Colon length shortening as a common parameter of colitis was estimated by measuring the dissected colons from each group. Analogously, colons of disease controls (G2) were the shortest, whereas in groups pretreated with phytoncide, colon length was less shortened in comparison with G2 (Figure 4B,C). Groups pretreated with phytoncide had lower DAI scores, thus showing its anti-inflammatory effects (Figure 4D). There was a slight, but not significant, difference between the prevention and therapy groups.

#### 6.3.2. Phytoncide Treatment Effects on Histological Changes in DSS-Induced Colitis

**Figure 5:**
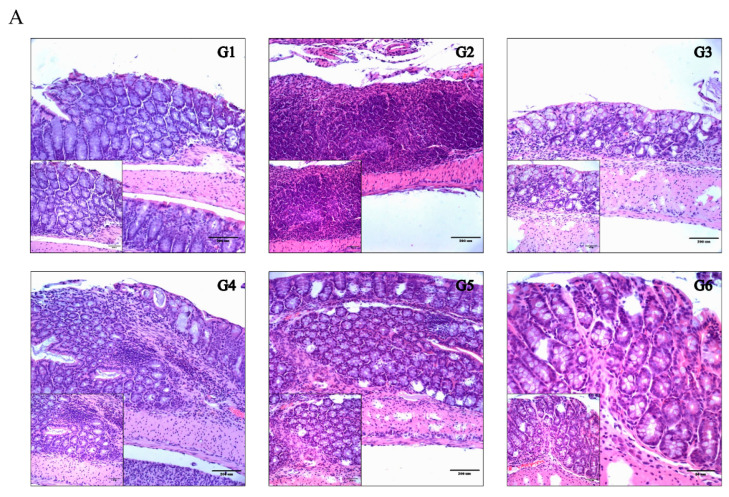

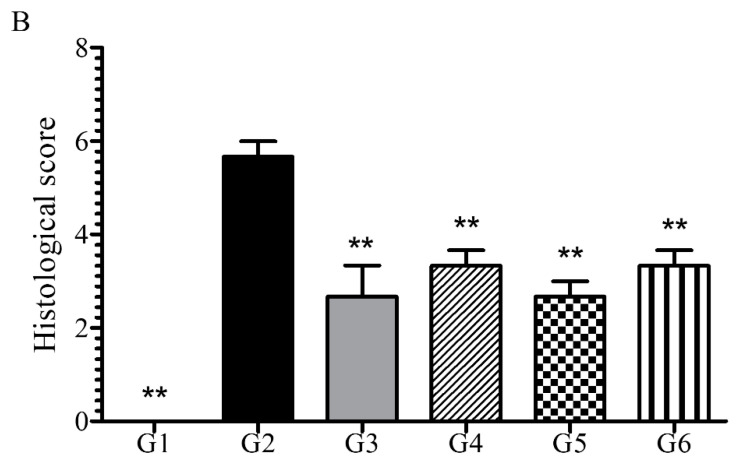
Effect of phytoncide treatment on histological changes on DSS-induced colitis damage. (**A**) Results of the H&E staining, × 100 magnification. (**B**) Effect of phytoncide on histological scores of DSS-induced colitis. G1, normal control; G2, vehicle control (colitis control, DSS 2% in DW); G3, prevention phytoncide oil 10 mL/kg; G4, therapy phytoncide oil 10 mL/kg; G5, prevention pine cone water extract 10 mL/kg; G6, therapy pine cone water extract 10 mL/kg. Representative data are expressed as mean ± SEM of experiments (*n* = 10) for each group. ** *p* < 0.01 compared to vehicle control.

To observe the histological change in colon tissue that occurs with DSS-induced colitis, H&E staining was performed. As shown in Figure 5A, colons from normal controls (G1) had a continuous epithelium surface with normal tubular glands. In the vehicle control group (G2), the distinct destruction of mucosal epithelium, crypt, and massive lesions, as well as infiltration of neutrophils and necrosis of cells, was observed. However, the PO- and PW-treated groups showed less destruction in comparison with G2. We scored these histological changes and confirmed that there was a significant difference between the groups (Figure 5B). In particular, the timing of phytoncide treatment (prevention or therapy) was more important than the type of phytoncide extract (PO or PW). The lesions were more alleviated in the prevention group (G3 and G5) than in the therapy group (G4 and G6) (*p* < 0.05). Given the histological changes, it can be concluded that phytoncide treatment can alleviate DSS-induced colitis in mice.

#### 6.3.3. Phytoncide Treatment Suppresses iNOS Expression in DSS-Induced Colitis

**Figure 6:**
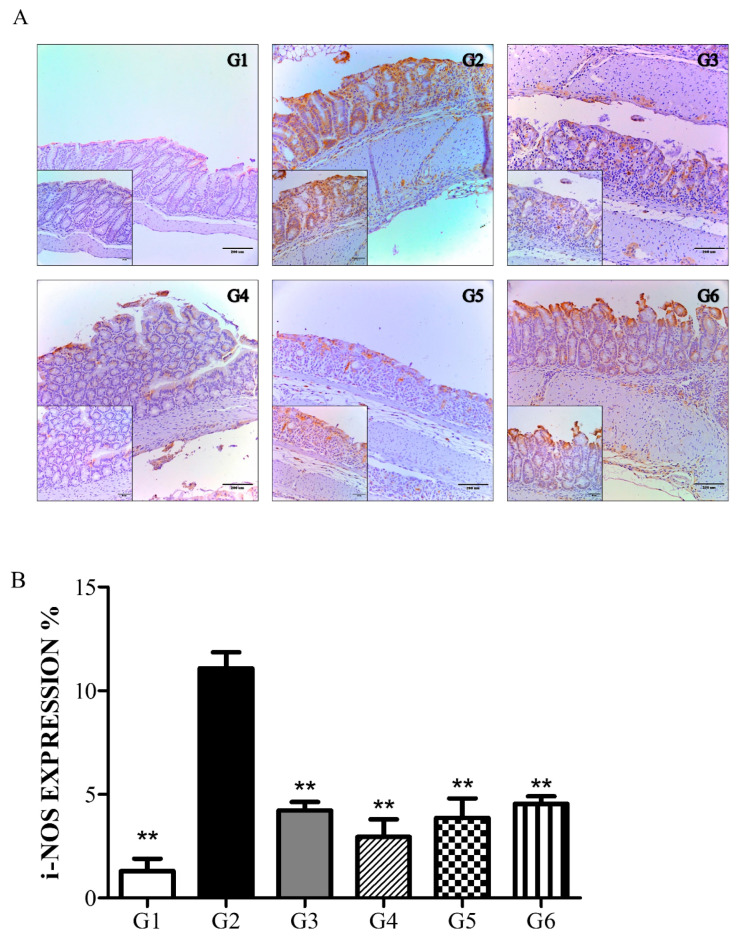
Result of immunohistochemical analysis: effect of phytoncide treatment on iNOS expression in DSS-induced colitis damage. (**A**) Photograph of colon from mouse with DSS-induced colitis, × 100 magnification. (**B**) iNOS expression graph of mouse with DSS-induced colitis. G1, normal control; G2, vehicle control (colitis control, DSS 2% in DW); G3, prevention phytoncide oil 10 mL/kg; G4, therapy phytoncide oil 10 mL/kg; G5, prevention pine cone water extract 10 mL/kg; G6, therapy pine cone water extract 10 mL/kg. ** *p* < 0.01 compared to vehicle control.

The level of iNOS expression in the colon was measured by immunohistological staining and then analyzing the brown-colored areas on the microphotographs. As shown in Figure 6, the normal control group (G1) showed a low level of iNOS expression, whereas in the vehicle control group (G2), it was markedly elevated. Greater down-regulation of iNOS expression was indicated in the PO and PW groups compared to G2. Similar to the histological score comparison (Figure 5B), the prevention groups seemed to show better suppression of iNOS expression than the therapy groups, but there was no significant difference. Ultimately, phytoncide treatment led to greater down-regulation of iNOS compared to that seen in the vehicle control group (G2).

## 7. Discussion

A recent study showed that alternative and complementary medicines have been used for managing the inflammatory system, which improves the disease condition by preventing inflammation [31]. Phytoncides have been reported to possess volatile organic compounds as natural antibacterial substances, and phytoncide includes numerous components such as α-pinene, carene, and myrcene [27]. In particular, one of the components of α-pinene is considered to be an anti-inflammatory substance. Phytoncide is being exploited for a variety of applications: as a deodorant, cholesterol inhibitor, anti-cancerous factor, and enhancer of human natural killer cell activity [29]. Additionally, phytoncide was revealed to have an antioxidant effect [28]. Phytoncide was chosen as a natural product to determine its anti-inflammatory effect in gastrointestinal diseases. We aimed to show that phytoncide’s anti-inflammatory influence on an animal’s digestive tract can be considered a first discovery that can lead to its use as a natural anti-inflammatory medicine in the future. The present experiment uses two classic representative methods for inducement of the inflammatory process in rodents.

The IM-induced gastric ulcer in rats was aggravated by oral administration of IM at a dose of 80 mg/kg [32]. IM is one of the most widely used NSAIDs, owing to its strong analgesic, antipyretic, and anti-inflammatory effects. However, it is also well known that IM has strong damaging effects on gastric mucosa [33]. NSAIDs have a direct effect on the gastric mucosa and can stop oxidative phosphorylation, which reduces electron transport in the mitochondrial membrane and leads to the release of cytochrome c. The released cytochrome c induces apoptosis by producing an oxygen radical, which plays an important role in protease activation [34,35]. Therefore, we wanted to see if phytoncide could act as a potent antioxidant and anti-inflammatory agent to suppress oxidative stress, which plays an important role in gastric mucosal damage by NSAIDs. In this study, it was confirmed that IM induced gastric ulcers, and at the same time, when PO and PW were provided, the mucosal tissue changes were significantly alleviated (*p* < 0.05). In particular, the anti-inflammatory effect of phytoncide was demonstrated by confirming that expression of iNOS, which mediates pro-inflammatory cytokine expression, dramatically decreased in the phytoncide-treated group.

Another part of the experiment focused on the mouse model of acute colitis using 2% DSS. This model resembles human ulcerative colitis, with a number of symptoms such as mucosal inflammatory cellular infiltration, ulceration, and epithelial damage [31,32]. Mice that drank water with DSS efficiently developed lesions in the colon, distinguished by diarrhea, bloody stools, shortening of colon length, and body weight loss [36]. We confirmed the anti-inflammatory effect of phytoncide on the colon. Macroscopic analyses showed disease development as well as clear signs of colitis in the DSS-induced colitis model. The animals in the phytoncide-treated groups clearly exhibited decreased shortening of the colon length. The DAI, which is used to assess the clinical pathogenesis of colitis by measuring its main physiological symptoms, decreased by 25% in the pretreated groups compared to the vehicle control group. Analysis of microscopic pictures of H&E-stained tissue samples and calculation of histological scores showed significant inhibition of inflammatory changes in the pretreated groups.

According to previously reported data, the inflammatory process in the digestive tract is associated with escalated iNOS expression and increased infiltration of leukocytes [37]. The NO and iNOS are involved in healing inflammatory tissue damage of the digestive tract [38] and play a critical role in colorectal carcinogenesis. Recently, IM-induced gastric ulcer development was associated with inhibition of NO genesis [39]. NSAID-induced gastric ulcers developed when COX-2 and iNOS-mediated PG synthesis were inhibited [40]. Moreover, DSS-induced colitis occurred with suppression of iNOS-mediated PG synthesis in mice [36]. As NO is a mediator of mucosal membrane defense, iNOS expression was measured to determine the anti-inflammatory effect [41]. In this study, the inhibition of iNOS expression with intragastric administration of phytoncide reached almost 70%, with a slight difference between the concentrations of phytoncide used in the pretreated groups in both the gastric ulcer model experiment and the colitis experiment.

In conclusion, our findings confirm that phytoncide treatment possesses anti-inflammatory properties in both the stomach and colon. Furthermore, it is likely that phytoncide treatment can heal not only the stomach and colon but also other parts of the digestive tract such as the esophagus, duodenum, and small intestine. This study can serve as a basic step to further, similar experiments for testing natural products expected to possess an anti-inflammatory effect on diseases of the digestive tract.

## Figures and Tables

**Table 1:** Conditions of GC-MS analysis for phytoncide obtained by steam distillation.

Instrument	Agilent Technologies 7890A, 5975C
Column	HP-5MS Column (30 m × 0.25 mm, 0.25 μm)
MS ion sourcetemperature	320 °C
Injection porttemperature	280 °C
Carrier gas	He
Column flow	1.5 mL/min
Split ratio	Split mode 10:1
Injection volume	1 μL
Oven temp.	50 °C for 3 min→raised to 150 °C at 2 °C/min→raised to 300 °C at 20 °C/min, held for 10 min
Ionization energy	70 eV
Selected ion	α-Pinene: SIM mode m/z 77. 91. 93. 121 Scan mode 40–600 amu

**Table 2:** Composition of phytoncide in (**A**) oil phase (PO) and in the (**B**) water phase (PW) through GC-MS analysis.

**A**	**No.**	**Retention Time (min)**	**Name**	**Area (%)**
	1	8.392	Alpha-Pinene	49.431
	2	8.942	Camphene	1.442
	3	10.007	Beta-Pinene	13.247
	4	10.697	Beta-Myrcene	3.689
	5	11.189	Bicyclo(4.1.0) hept-3-ene	3.067
	6	11.997	Limonene	27.118
	7	23.280	1,4-Methanoazulene	1.217
	8	23.572	Caryophyllene	0.789
**B**	**No.**	**Retention Time (min)**	**Name**	**Area (%)**
	1	18.96	Verbenone	9.230
	2	18.66	Alpha-terpinieol	8.110
	3	16	Fenchol	6.830
	4	14.36	Camphor	4.750
	5	18.74	Borneol	4.660
	6	12.35	*p*-Alpha-dimethylstyrene	4.430
	7	20.62	Myrtenol	3.970
	8	21.46	Carveol	3.860

## Data Availability

Not applicable.

## Sample Availability

Samples of the compounds are NOT available from the authors.
